# Reversal of Paraneoplastic Non-Bacterial Thrombotic Endocarditis with Heparin and Targeted Cancer Therapy: A Case Report

**DOI:** 10.3390/reports9010074

**Published:** 2026-02-28

**Authors:** Collin Goetze, Nikolaj Frost, Ingo Hilgendorf, Daniel Armando Morris, Matthias Schneider-Reigbert

**Affiliations:** 1 Department of Cardiology, Angiology and Intensive Care Medicine, Deutsches Herzzentrum der Charité (DHZC), Charité—Universitätsmedizin Berlin, 13353 Berlin, Germany; 2 DZHK (German Center for Cardiovascular Research), 10785 Berlin, Germany; 3 Faculty of Medicine, Charité—Universitätsmedizin Berlin, Corporate Member of Freie Universität Berlin, Humboldt-Universität zu Berlin, and Berlin Institute of Health, 10117 Berlin, Germany; 4 Department of Infectious Diseases and Pulmonary Medicine, Charité—Universitätsmedizin Berlin, 13353 Berlin, Germany; 5 National Center for Tumor Diseases (NCT), 13353 Berlin, Germany

**Keywords:** non-bacterial thrombotic endocarditis, lung adenocarcinoma, rivaroxaban, low-molecular-weight heparin, cancer-associated thrombosis, targeted therapy

## Abstract

**Background and Clinical Significance:** Non-bacterial thrombotic endocarditis (NBTE), historically termed marantic endocarditis, is a severe manifestation of cancer-associated hypercoagulability characterized by sterile valvular vegetations and a high risk of systemic embolization. While direct oral anticoagulants (DOACs) have become the standard of care for cancer-associated venous thromboembolism (CAT), their efficacy in preventing high-shear arterial thrombosis in NBTE has been contested. Emerging data suggest that DOACs may fail to halt vegetation growth in active malignancy, necessitating a reversion to heparin-based therapies. **Case Presentation:** A 47-year-old female with metastatic RET fusion-positive non-small cell lung cancer (NSCLC) presented with progressive dyspnea and digital ischemia despite strict adherence to therapeutic anticoagulation with rivaroxaban for a prior pulmonary embolism. Echocardiography showed large vegetations on all three cusps of the aortic valve, confirming NBTE. Computed tomography revealed extensive tumor progression. The therapeutic strategy involved an immediate switch from rivaroxaban to therapeutic low-molecular-weight heparin (LMWH) and the initiation of dual targeted therapy with selpercatinib and tepotinib. Serial transesophageal echocardiography documented regression within two weeks and eventual complete resolution of the valvular vegetations after eight weeks, occurring in tandem with a rapid radiological response of the tumor. **Conclusions:** Upon diagnosis of NBTE, a rapid oncologic work-up is warranted, as ongoing tumor progression is highly likely. This case questions the appropriateness of direct oral anticoagulants in patients with NBTE and active, progressive malignancy.

## 1. Introduction and Clinical Significance

Non-bacterial thrombotic endocarditis (NBTE) is a rare but devastating cardiovascular manifestation of advanced malignancy and represents an extreme phenotype of cancer-associated hypercoagulability. The condition is characterized by sterile platelet–fibrin thrombi that preferentially form on cardiac valves, most commonly the aortic and mitral valve, in the absence of microbial infection [1]. Its pathophysiology is driven by a paraneoplastic prothrombotic state mediated by tumor-derived tissue factor, circulating mucins, inflammatory cytokines, and endothelial activation [2]. Clinically, NBTE is associated with a high risk of systemic embolization, including ischemic stroke and peripheral arterial occlusion, and carries a poor prognosis if not recognized and treated promptly [3].

Despite increasing recognition of NBTE, optimal anticoagulation strategies remain a subject of ongoing debate. Over the past decade, direct oral anticoagulants (DOACs) such as rivaroxaban and apixaban have largely replaced low-molecular-weight heparin (LMWH) in the management of cancer-associated venous thromboembolism, based on randomized trials demonstrating non-inferiority and improved convenience in venous disease [4,5,6]. However, these data cannot be readily extrapolated to NBTE, which represents an arterial, valve-based thrombotic process occurring under high-shear conditions. Emerging clinical observations and mechanistic considerations suggest that DOACs may be less effective in this setting, where platelet–endothelial interactions and inflammation play a central role [7].

Heparins exert biological effects that extend beyond factor Xa inhibition, including interference with platelet adhesion, modulation of endothelial activation, and attenuation of inflammatory signaling [8]. These pleiotropic properties may be particularly relevant in NBTE, where sterile vegetations are sustained by continuous paraneoplastic procoagulant and inflammatory stimuli [9]. Nevertheless, robust comparative data are lacking, and clinical decision-making is often guided by case reports and expert consensus rather than high-level evidence.

We report the case of a 47-year-old patient with metastatic RET (rearranged during transfection) fusion non-small cell lung cancer (NSCLC) who developed severe aortic valve NBTE while receiving therapeutic rivaroxaban. This case emphasizes the considerable variability in the progression of NBTE. Complete resolution occurred within weeks after switching to LMWH. A similar response has previously been reported by our group in stable oncologic disease [10]. In the current patient, targeted oncologic therapy with rapid tumor regression was initiated almost simultaneously with the switch to LMWH. In addition to anticoagulation, the activity of the underlying malignancy may influence the persistence and recurrence of NBTE. Tumor burden may act as a continuous driver of systemic hypercoagulability, raising the question of whether effective oncologic disease control can modulate thrombotic risk once adequate anticoagulation is established [11]. However, the relative contribution of tumor regression versus anticoagulant choice remains uncertain and controversial.

**Clinical Significance.** This case describes the development of severe aortic valve NBTE despite therapeutic anticoagulation with rivaroxaban in a patient with metastatic RET-rearranged non-small cell lung cancer. It underscores the need for prompt oncologic reassessment once NBTE is diagnosed, as this condition typically reflects highly progressive malignancy. In line with previous reports, the case again documents failure of direct oral anticoagulant therapy in a high-risk paraneoplastic setting. Complete resolution of valvular vegetations was observed within weeks after switching to LMWH; however, concomitant tumor regression occurred during the same period, leaving uncertainty as to whether NBTE regression was driven primarily by anticoagulation strategy or by oncologic disease control.

## 2. Case Presentation

### 2.1. Patient Information and Clinical Findings

A 47-year-old woman with stage IVB lung adenocarcinoma was admitted to our center on 9 July 2025 because of rapidly progressive dyspnea (CTCAE grade 3), dizziness, and right-sided thoracic pain. The initial cancer diagnosis had been established in February 2024, revealing a KIF5B–RET fusion-positive tumor. First-line therapy with selpercatinib (160 mg daily) was initiated in March 2024, achieving an initial partial response. Following multifocal progression in November 2024, which required palliative radiotherapy for osseous metastases, re-biopsy revealed an acquired MET amplification. Consequently, the treatment was switched to second-line chemotherapy with cisplatin and pemetrexed in December 2024, followed by pemetrexed maintenance starting in March 2025. Apart from the underlying malignancy and its related complications, the patient had no known history of cardiovascular disease or other pre-existing thromboembolic conditions. At the time of diagnosis, the disease course was complicated by extensive venous thromboembolism, including bilateral pulmonary embolism and deep vein thrombosis, for which therapeutic anticoagulation with rivaroxaban (20 mg once daily) had been initiated. The patient reported strict adherence to the prescribed regimen.

On admission, she appeared markedly debilitated (Eastern Cooperative Oncology Group (ECOG) performance status 3), with resting tachycardia and mild hypoxemia (peripheral oxygen saturation (SpO_2_ 92%) requiring supplemental oxygen. Pulmonary examination demonstrated markedly reduced breath sounds over the right hemithorax, consistent with a large pleural effusion. Cardiac auscultation revealed regular heart sounds without audible murmurs. Examination of the extremities revealed a painful livid discoloration of the left hallux (“blue toe”), suggestive of peripheral microembolization.

### 2.2. Diagnostic Assessment

Laboratory testing showed pronounced systemic inflammation with markedly elevated C-reactive protein (CRP) levels (peak 435 mg/L) and increased D-dimer concentrations, consistent with a hyperinflammatory and hypercoagulable state. Repeated peripheral and central blood cultures remained negative, and serologic testing for atypical pathogens associated with infective endocarditis was unremarkable.

**Figure 1:**
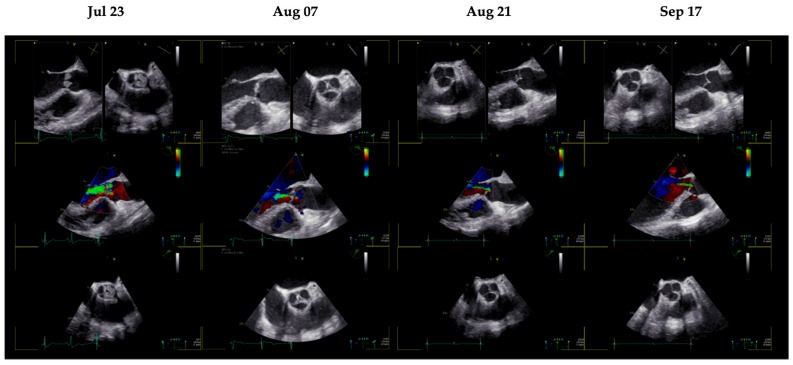
TEE examinations from initial diagnosis (23 July 2025) until complete resolution (17 September 2025). Significant regression is visible after 2 weeks of LMWH and targeted therapy (7 August 2025), with near-complete resolution by week 4 (21 August 2025), and confirmed complete resolution after 8 weeks (17 September 2025). TEE: transesophageal echocardiography. LMWH: low-molecular-weight heparin.

Given the presence of digital ischemia, an embolic source was suspected. Transthoracic echocardiography raised concern for valvular pathology, which was confirmed by TEE performed on 23 July 2025. TEE demonstrated echodense vegetations on all three cusps of the aortic valve, associated with moderate-to-severe aortic regurgitation, representing the “marantic kiss” and “marantic star” signs (Figure 1) [12]. Quantification of hemodynamic parameters revealed preserved left ventricular function (LVEF 60%) with no echocardiographic evidence of aortic stenosis (mean pressure gradient 4 mmHg). No intracardiac thrombi were detected. In the absence of microbiological evidence of infection, the diagnosis of NBTE was established, notably occurring despite ongoing therapeutic anticoagulation with rivaroxaban.

Cross-sectional imaging demonstrated marked oncologic disease progression. A computed tomography (CT) scan performed on 1 July 2025 revealed substantial progression of bilateral pulmonary tumor burden and hepatic metastases, with the largest hepatic lesion measuring 12.8 cm.

### 2.3. Therapeutic Intervention

A multimodal treatment strategy was implemented, addressing anticoagulation failure, oncologic disease activity, and symptom control.

- Anticoagulation: Rivaroxaban was discontinued. Anticoagulation was switched to therapeutic LMWH (nadroparin 0.8 mL twice daily).
- Targeted oncologic therapy: To address both the primary RET fusion and the acquired MET (mesenchymal–epithelial transition) amplification resistance mechanism, combined treatment with selpercatinib (RET inhibitor, continued at 160 mg twice daily) and tepotinib (MET inhibitor, 450 mg once daily) was initiated on 17 July 2025.
- Supportive measures: A pleural catheter was placed for management of the malignant pleural effusion. Empiric broad-spectrum antibiotic therapy (vancomycin and cefotaxime) was started initially but discontinued once infective endocarditis had been reliably excluded.

### 2.4. Follow-Up and Outcomes

**Figure 2:**
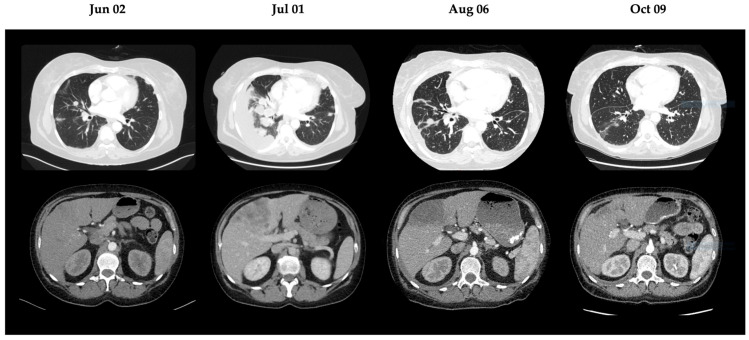
Radiologic evolution of metastatic disease. Serial CT scans of the thorax (top) and abdomen (bottom) demonstrate extensive progression of pulmonary and hepatic metastases on 1 July 2025, followed by rapid regression on 6 July 2025 and 9 October 2025 after initiation of dual targeted therapy. CT: computed tomography.

Clinical improvement was observed within weeks of treatment modification. A restaging computed tomography (CT) scan on 6 August 2025 demonstrated regression of pulmonary and hepatic metastases, as shown in Figure 2. Serial echocardiographic follow-up revealed progressive improvement of valvular findings. A TEE performed on August 7th (week 2) showed partial regression of aortic valve vegetations, and by August 21st (week 4), only a single residual vegetation remained on the right coronary cusp. Subsequent TEE examinations confirmed complete resolution of valvular vegetations by September 2025 (week 8), as can be seen in Figure 1.

**Figure 3:**
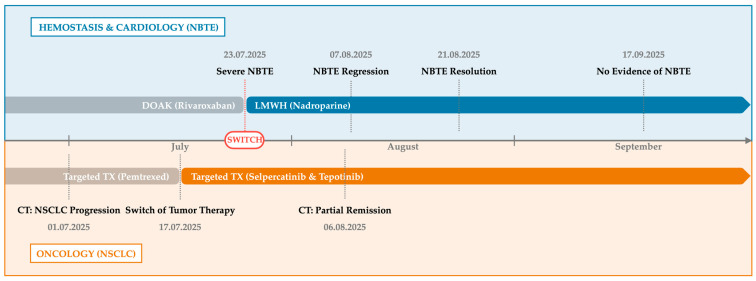
Integrated timeline of clinical management and disease response. The switch from DOAC to LMWH combined with targeted therapy led to rapid regression of aortic valve vegetations and tumor burden, with complete resolution of NBTE by week 8. DOAC: direct oral anticoagulant, LMWH: low-molecular-weight heparin, NBTE: non-bacterial thrombotic endocarditis.

The patient was discharged in stable condition. An outpatient TEE on 17 September 2025 confirmed sustained absence of valvular lesions. Digital ischemia resolved with conservative management. At the last follow-up in December 2025, the patient remained clinically stable under continued anticoagulation with LMWH and ongoing targeted oncologic therapy. An integrated timeline of diagnostic findings, therapeutic interventions, and clinical outcomes is presented in Figure 3 and summarized in Table 1.

## 3. Discussion

This case highlights the unique pathophysiological challenges of managing paraneoplastic thrombosis and offers critical insights into the limitations of DOACs in high-shear arterial flow.

**Mechanisms of DOAC Failure in NBTE.** The development of aortic valve vegetations and digital ischemia while on therapeutic rivaroxaban suggests a fundamental inadequacy of factor Xa inhibition in the context of NBTE. Unlike venous thromboembolism, which is primarily driven by stasis and fluid-phase coagulation factors, NBTE vegetations are platelet-rich thrombi formed in the high-velocity, high-shear environment of the arterial circulation [1,13]. In mucin-producing adenocarcinomas, tumor cells shed tissue factor-bearing microparticles and mucins that can directly activate platelets and factor X, often overwhelming the stoichiometric inhibition provided by standard DOAC dosing. Furthermore, DOACs lack the non-anticoagulant properties required to disrupt the initial adhesion of these microparticles to the valvular endothelium [2,7,9,13,14].

**Biological Advantages of LMWH in NBTE.** The resolution of valvular vegetations following the switch to nadroparin supports the use of LMWH as the preferred anticoagulant in NBTE [6,7,10]. Beyond its anticoagulant effect, heparin exerts several biologically relevant actions that are particularly important in this setting. Heparin binds to P-selectin, thereby reducing platelet and leukocyte adhesion to the endothelium—an early and critical step in the formation of sterile vegetations [8]. In addition, heparin neutralizes cationic inflammatory mediators, including chemokines and cytokines, and modulates tissue factor pathway inhibitor (TFPI)-related pathways, resulting in a local anti-inflammatory effect at the valve surface [15]. Together, these anti-adhesive, anti-inflammatory, and antithrombotic properties provide a multi-level protection against paraneoplastic thrombogenesis that cannot be achieved by small-molecule anticoagulants such as rivaroxaban.

**Tumor Regression as a Potential Contributor to NBTE Resolution.** While LMWH has previously been shown to improve NBTE, this case also raises the possibility that concurrent tumor control may have contributed to the sustained resolution of vegetations. The underlying malignancy likely served as a persistent prothrombotic driver, maintaining a state of systemic hypercoagulability through ongoing release of tumor-associated pro-coagulant factors [11]. Notably, the complete disappearance of valvular vegetations occurred in parallel with rapid radiological regression of hepatic and pulmonary metastases following (re-) initiation of selpercatinib and tepotinib. Although this temporal association does not establish causality, it suggests that effective molecular therapy may reduce the thrombotic milieu and thereby facilitate endogenous fibrinolysis once adequate anticoagulation with LMWH is in place.

**Clinical Implications.** Clinicians must maintain a low threshold for suspecting NBTE in cancer patients with new embolic events, even those already anticoagulated. This case serves as a warning signal that, despite the convenience of oral anticoagulation, the specific pathophysiology of NBTE may require the pleiotropic effects of heparin for effective management. At the same time, diagnosis of NBTE should prompt oncological work-up with the suspicion of tumor progression.

## 4. Conclusions

This case demonstrates the failure of rivaroxaban to prevent paraneoplastic NBTE, reinforcing LMWH as the superior anticoagulant in this setting. The complete resolution of valvular vegetations, occurring in tandem with rapid tumor regression, supports that successful management requires synergistic heparin-based anticoagulation and aggressive molecular control of the malignancy.

## Figures and Tables

**Table 1:** Timeline of clinical events, diagnostic investigations, therapeutic interventions, and outcomes in a patient with non-bacterial thrombotic endocarditis associated with metastatic lung adenocarcinoma.

Date	Event/Investigation	Key Findings
	NSCLC diagnosis	Stage IVB lung adenocarcinoma; KIF5B–RET fusion-positive tumor, start selpercatinib
February 2024	Venous thromboembolism	Bilateral pulmonary embolism and deep vein thrombosis
	Anticoagulation initiated	Rivaroxaban 20 mg once daily
12 November 2024	CT thorax/abdomen	Progressive disease NGS testing: MET amplification as secondary resistance Start cisplatin/pemetrexed
1 July 2025	CT thorax/abdomen	Progression of pulmonary tumors and hepatic metastases
9 July 2025	Hospital admission	Dyspnea (CTCAE grade 3), pleural effusion, digital ischemia
17 July 2025	Targeted tumor therapy	Selpercatinib + tepotinib (off-label combination)
23 July 2025	TEE	Diagnosis of NBTE under DOAC therapy
	Anticoagulation switch	Rivaroxaban discontinued; nadroparin initiated
30 July 2025	TEE	Echocardiographic NBTE unchanged
6 August 2025	CT thorax/abdomen	Regression of pulmonary tumors and hepatic metastases
7 August 2025	TEE	Partial regression of aortic valve vegetations
21 August 2025	TEE	Single residual vegetation on the right coronary cusp
17 September 2025	TEE	Complete resolution of aortic valve vegetations
9 October 2025	CT thorax/abdomen	Further regression of pulmonary tumors and hepatic metastases
21 October 2025	TEE	Persistent absence of vegetations
18 December 2025	TEE	Persistent absence of vegetations

## Data Availability

The original contributions presented in this study are included in the article. Further inquiries can be directed to the corresponding author.

## Abbreviations

The following abbreviations are used in this manuscript:

NBTE	non-bacterial thrombotic endocarditis
DOACs	direct oral anticoagulants
LMWH	low-molecular-weight heparin
NSCLC	Non-small cell lung cancer
CTCAE	Common Terminology Criteria for Adverse Events
ECOG	Eastern Cooperative Oncology Group
SpO_2_	peripheral oxygen saturation
CRP	C-reactive protein
TEE	transesophageal echocardiography
CT	computed tomography
RET	rearranged during transfection
MET	mesenchymal–epithelial transition
TFPI	tissue factor pathway inhibitor
